# Ultrasound-Guided Pudendal Nerve Block Combined with Propofol Deep Sedation versus Spinal Anesthesia for Hemorrhoidectomy: A Prospective Randomized Study

**DOI:** 10.1155/2021/6644262

**Published:** 2021-02-26

**Authors:** Jian He, Lei Zhang, Dong L. Li, Wan Y. He, Qing M. Xiong, Xue Q. Zheng, Mei J. Liao, Han B. Wang

**Affiliations:** Department of Anesthesiology, The First People's Hospital of Foshan, Foshan, China

## Abstract

*Background and Objectives. *Several anesthesia techniques were applied to hemorrhoidectomy, but postoperative pain and urinary retention were still two unsolved problems. The aim of this prospective randomized study was to evaluate the effect of ultrasound-guided pudendal nerve block (PNB) combined with deep sedation compared to spinal anesthesia for hemorrhoidectomy. *Methods*. One hundred and twenty patients undergoing Milligan–Morgan hemorrhoidectomy were randomized to receive PNB combined with deep sedation using propofol (Group PNB, *n* = 60) or spinal anesthesia (Group SA, *n* = 60). Pain intensity was assessed using the visual analogue scale (0: no pain to 10: worst possible pain). The primary outcome was pain scores recorded at rest at 3, 6, 12, 24, 36, and 48 h and on walking at 12, 24, 36, and 48 h postoperatively. Secondary outcomes were analgesic consumption, side effects, and patient satisfaction after surgery. *Results*. Ultrasound-guided bilateral PNB combined with deep sedation using propofol could successfully be applied to Milligan–Morgan hemorrhoidectomy. Postoperative pain intensity was significantly lower in Group PNB compared to Group SA at rest at 3, 6, 12, 24, 36, and 48 h (*p* < 0.001) and during mobilization at 12, 24, 36, and 48 h (*p* < 0.001) postoperatively. Sufentanil consumption in Group PNB was significantly lower than that in Group SA, during 0–24 h (*p* < 0.001) and during 24–48 h (*p* < 0.001) postoperatively. Urinary retention was significantly lower in Group PNB compared to Group SA (6.9% vs 20%, *p*=0.034). The patients in Group PNB had higher satisfaction compared to Group SA (*p* < 0.001). *Conclusions*. Ultrasound-guided PNB combined with propofol sedation is an effective anesthesia technique for Milligan–Morgan hemorrhoidectomy.

## 1. Introduction

Hemorrhoidal disease prevails in all mankind ages. Hemorrhoidectomy is one of the best methods to cure serious hemorrhoidal disease, providing a good quality of life [1]. Among the various techniques for hemorrhoidectomy, Milligan–Morgan hemorrhoidectomy is still the “gold standard” treatment, since it is the most radical one with superior outcome [1, 2]. Several anesthesia techniques have been successfully used for hemorrhoidectomy, including general anesthesia, spinal anesthesia, and local infiltration blocks [3–6]. However, postoperative pain and urinary retention are still two main unsolved problems after the operation. Previous studies have indicated that 20%–40% of patients who underwent hemorrhoidectomy would endure severe postoperative pain, even in analgesic therapy [7, 8]. Lumbar epidural analgesia has been reported to produce effective pain relief after hemorrhoidectomy, but it often leads to urinary retention. Moreover, epidural or spinal anesthesia is the main factor of urinary retention after surgery [3]. Local infiltration anesthesia can provide certain postoperative analgesia without increasing the incidence of urinary retention, but it cannot provide complete muscle relaxation [9]. In recent years, spinal anesthesia combined with pudendal nerve block (PNB) has been performed for hemorrhoidectomy and it could decrease postoperative pain [10]. However, this anesthesia technique could not decrease the incidence of postoperative urinary retention. Otherwise, PNB alone is not enough relaxation for hemorrhoidectomy, increases the difficulty of operation, and may cause patient discomfort. The ideal anesthesia technique for hemorrhoidectomy should provide relatively prolonged pain relief without increasing postoperative urinary retention and provide competent anal sphincter muscle relaxation.

The pudendal nerve originates from sacral nerves S2, S3, and S4 and innervates the perineal and perianal skin sensation and muscle motor [11]. In theory, PNB enables analgesia or anesthesia of the perineal region. Previous studies reported that PNB can provide excellent postoperative analgesia for hemorrhoids surgery [5, 9, 11–14]. The PNB was recommended for all patients undergoing hemorrhoidal surgery by PROSPECT (PROcedure-SPECific postoperative pain managemenT) Working Group [15]. Tepetes et al. [5] indicated that PNB was more efficient than perianal local anesthetic infiltration at reducing pain and the need for analgesics. However, the use of PNB alone could not provide complete puborectalis muscle relaxation or lead to the patient's discomfort during the operation. Deep sedation may reduce the patient's discomfort and vagal reflex.

This study aimed to investigate whether PNB combined with deep sedation using propofol could be successfully applied to Milligan–Morgan hemorrhoidectomy and decrease postoperative pain and the incidence of urinary retention compared to spinal anesthesia.

## 2. Methods

The study was approved by the China Ethics Committee of Registering Clinical Trials (ChiECRCT-20180226) before the first patient recruitment. It was registered in the China Clinical Trial Registry on January 24, 2019 (Registration number: ChiCTR1800020162). All patients gave written informed consent before inclusion in the study. This single center, prospective, randomized, parallel-group study was carried out in the First People's Hospital of Foshan in China from February 23, 2019, to September 25, 2019 (last patient follow-up). The study protocol was not changed after the start of the research. Patients with American Society of Anesthesiologists (ASA) status I-II, aged 18–75 years, submitted to elective Milligan–Morgan hemorrhoidectomy were included in this study. Exclusion criteria were as follows: patients with chronic pain; vertebral and pelvic deformities; history of allergy to any drugs used in the study; hemorrhagic diseases; severe liver, kidney, or heart diseases; anal fistula and fissure; mental illness; obesity; apnea distress syndrome; pregnancy and lactation; and inability to understand and use a patient-controlled pump for analgesia. All patients stopped taking nonsteroidal anti-inflammatory drugs or acetylsalicylic acid prior to surgery. Patients were given instructions about the visual analogue scale (VAS) for pain assessment, with scores ranging from 0 to 10 (0 = no pain, 10 = worst imaginable pain), and the use of the patient-controlled analgesia (PCA) device prior to anesthesia.

Randomization was performed by concealed allocation using a computer-based (https://www.randomizer.org/) random number to generate a randomization list; these were inserted into sequentially numbered, opaque, sealed envelopes. A total of 120 consecutive numbered envelopes (60/group) were thus made by statistical personnel with no further involvement in this study. The randomization list was stored in a locked iron sheet cabinet and was not accessible to the staff engaged in the study. Subjects included in the study were assigned to receive anesthesia methods based on the randomization list. Allocation concealment was opened only after the participant was enrolled in the study. Data collection after surgery was performed by a staff member who was blinded to the grouping.

### 2.1. Anesthesia and Surgery

All patients underwent routine preanesthetic evaluation and received standard monitors, including heart rate (HR), noninvasive blood pressure, pulse oxygen saturation (SpO_2_), and electrocardiography. The patients were given oxygen by a face mask at the rate of 2 liters min^−1^. After the peripheral venous access was established, Ringer's lactate was infused continuously during the operation. A limited fluid infusion strategy was implemented, and the fluid volume was not more than 500 ml during the surgery. Five micrograms of sufentanil was intravenously injected for analgesia and sedation before spinal anesthesia or pudendal nerve block.

The patients from Group SA received spinal anesthesia with left lateral decubitus, in the space L3-L4, using the median approach with a 25 G spinal needle. Once free flow of cerebrospinal fluid was confirmed, 2.0–2.5 ml of 0.5% ropivacaine (heavy), based on the patient demographic data, was administered. Then, the patient was subsequently made supine. Sedation was maintained by 0.5 microg.kg^−1^.h^−1^ dexmedetomidine continuous intravenous infusion without a loading dose.

**Figure 1:**
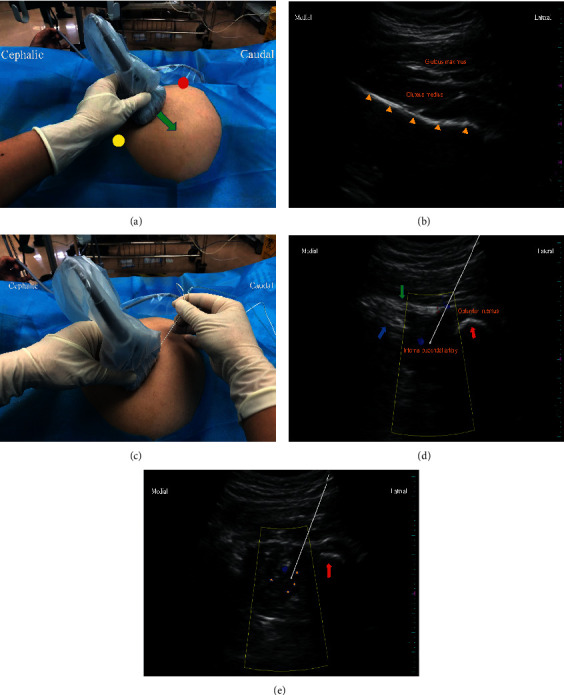
The ultrasound prober location and gray-scale and color Doppler images. (a) The ultrasound transducer is placed at the midpoint of the line connecting the greater trochanter (red circle) and the posterior superior iliac spine (yellow circle). The green arrow indicates the direction of ultrasound transducer movement. (b) The ultrasound image when the transducer is placed at the midpoint of the line connecting the greater trochanter and the posterior superior iliac spine. Hip bone is seen as hyperechoic specular reflectors (yellow triangle). (c) The final position of ultrasound transducer placed and a lateral to medial approach injection. (d) The ultrasound image when the transducer shifts to the lesser sciatic notch. Blue and green arrows indicate sacrospinous and sacrotuberous ligaments, respectively. The red arrow indicates ischial tuberosity. The internal pudendal artery (blue Doppler signal) lies in the corner between the coccygeus and internal obturator muscles. The white arrow indicates needle trajectory and injection point. (e) The ultrasound image after anesthetic injection around the internal pudendal artery. The pentagram represents the spread of local anesthetics.

Patients from Group PNB received bilateral PNB with 30 ml of 0.4% ropivacaine containing 0.5 microg.kg^−1^ dexmedetomidine, 15 ml for each side. PNB was performed by the same anesthesiologist (J.H.) as a single injection in the preanesthesia room. The anesthesiologist had performed more than 50 pudendal nerve blocks before participating in this study. According to a previous study [16], pudendal nerve was blocked at the entrance of the pudendal (Alcock) canal. With the patient in the lateral decubitus position, a low-frequency curvilinear transducer (2–5 MHz) was put on the midpoint of the line connecting the greater trochanter and the posterior superior iliac spine (Figure 1(a)). In this position, a continuous hyperechoic iliac bone line was identified by ultrasound (Figure 1(b)). Then, transducer was moved parallel and caudal to the lesser sciatic notch, and the pudendal nerve, artery, and vein were visualized inside the proximal part of the Alcock canal in the sharp angle between the coccygeus and internal obturator muscles (Figures 1(c) and 1(d)). The pulsation of the internal pudendal artery was visible with color Doppler on the internal surface of the internal obturator muscles inside the Alcock canal (Figure 1(d)). An 80 to 120 mm (dependent on patient size), 22 G needle (Stimuplex Ultra needle; B. Braun, Melsungen, Germany) was inserted in-plane from lateral to medial approach and advanced until the needle tip located close to the pudendal artery (Figures 1(d) and 1(e)). The procedure was performed in combination with electrical nerve stimulation as a safety measure to avoid intraneural injection, with a level of electrical stimulation of 0.2 mA, 100 milliseconds, and 2 Hz-typically without producing paresthesia in the perineum. Then, fifteen milliliters of 0.4% ropivacaine containing 0.5 microg.kg^−1^ dexmedetomidine was injected slowly with intermittent aspiration. The patient was changed to the other side lateral decubitus position for the contralateral pudendal nerve block by the same ultrasonic scanning technology. Thirty minutes later, if the sensation of the skin around the anus and the contractility of the anus decreased, this indicated that the pudendal nerve was successfully blocked. Target-controlled infusion of propofol combined with 3–5-microgram sufentanil intravenous injection was given for sedation in the PNB group. The initial plasma concentration of propofol was set to 2.0 microg.ml-1 and gradually increased by 0.5 microg.ml-1 each time, up to 4.5 microg.ml-1 plasma concentration. When the effect-site concentration of propofol for the loss of consciousness (LOC) of the patient was achieved and balanced, the surgeon was allowed to perform the surgery. After the anal dilator was inserted, the plasma concentration of propofol was reduced and maintained at 30% higher than the concentration of LOC. If body movement occurred during the surgery, the plasma concentration of propofol was increased. The plasma concentration of propofol was maintained at 2.5–4.5 microg.ml-1 throughout the operation.

The patients from the two groups underwent surgery in the left lateral decubitus position. According to a previously reported procedure [17], a standard Milligan–Morgan hemorrhoidectomy was conducted by the same surgical team.

### 2.2. Treatment of Respiratory Depression and Hypotension

The respiratory rate of patients was monitored by end-tidal carbon dioxide probe and electrocardiogram analysis. Respiratory depression was considered as respiratory frequency less than 8 times per minute or SpO_2_ less than 94%. When respiratory depression occurred, the plasma concentration of propofol was decreased and the patient jaw was lifted. If SpO_2_ still could not be maintained above 94%, general anesthesia was induced and a laryngeal mask was inserted for ventilation. Hypotension was defined as the systolic pressure decreased by more than 20% of the baseline value or less than 90 mmHg. If hypotension occurred, 6 mg ephedrine was intravenously administered.

### 2.3. Preoperative and Postoperative Pain Management

All patients received intravenous acetaminophen 1000 mg 1 hour prior to surgery. In the orthopedic ward, patients were administered acetaminophen 500 mg orally every 6 hours and received 10 mg sustained-release oxycodone p.o. every 12 hours after the operation until the patient was discharged from the hospital. Additionally, a PCA device was provided as rescue analgesia with 2-microgram intravenous sufentanil injection when required with a 10 min lock-out time, and the maximum dose was 6 micrograms per hour. After 48 hours, the PCA device was removed. This is the standard postoperative analgesia protocol used in our hospital.

### 2.4. Recordings and Measurements

All patients were instructed to assess the intensity of their pain using the VAS, with scores ranging from 0 (no pain) to 10 (the worst possible pain). The primary outcome was the VAS at rest at 3, 6, 12, 24, 36, and 48 h after surgery and on walking at 12, 24, 36, and 48 h postoperatively by two specially trained assistants (one nurse and one resident doctor). Secondary outcomes included the following:

- Incidence of urinary retention during 0–48 h after surgery (need for catheterization): The patient was unable to void with a bladder volume more than 600 ml by bedside ultrasound; a urinary catheter was inserted.
- Total sufentanil consumption by each patient during 0–24 h and 24–48 h after surgery.
- The time from the end of the surgery to the first request for analgesic from the PCA device.
- The time from the end of surgery to the first defecation, and pain intensity at the first defecation.
- Sphincter relaxation assessment: Two-finger dilatation was recorded by the same surgeon on the scale of 3: scale 1, not relaxed; scale 2, incompletely relaxed; and scale 3, fully relaxed, and the surgeon was not related to the study and the surgery. If the sphincter relaxation was assessed by the scale as 1, spinal anesthesia was administered.
- Surgeon satisfaction with operative conditions using the following scale: 1, terrible; 2, satisfactory; and 3, excellent.
- Hypotension and respiratory depression during operation.
- Side effects: Postoperative nausea and vomiting, pruritus, and respiratory depression, from 0 to 48 h after surgery.
- Patient satisfaction: This was assessed when the patient was discharged from hospital using the following scale: 1, terrible; 2, poor; 3, satisfactory; 4, good; and 5, excellent.
- Time to ambulation was defined as the time from the end of the surgery to the time when the patient could go to the toilet unaided.

### 2.5. Statistical Analysis

Our pilot study and a previous study [18] showed that the mean pain VAS score at 12 h was 5 (standard deviation [SD]: 3.5) in patients with hemorrhoidectomy under the spinal anesthesia. Utilizing *α* = 80% and *β* = 0.05, a 2-tailed analysis showed that we needed 49 patients/group for a reduction in pain intensity by 40% in the case of patients with hemorrhoidectomy under PNB with deep sedation. We planned to recruit a total of 120 patients to compensate for 20% dropouts. Continuous variables were summarized as mean and SD. Categorical data were described using frequencies or proportions. Statistically significant differences between the two randomized groups were compared using the independent *t*-test for normally distributed continuous outcome variables or Mann–Whitney *U* test for non-normally distributed continuous variables. Chi-square test or Fischer's exact test was used to compare the study groups for categorical data, such as side effects and complications. A repeated-measures analysis of variance (ANOVA) was used to estimate the difference in VAS scores between the two groups at each time point. *p*-values <0.05 were considered significant. All statistical analyses were performed using SPSS version 16. After completion of the study, the data were typed into a spreadsheet by two researchers. A randomization list assigning subjects to either Group “A” or “B” was created without revealing the identity of the groups. The statistical analysis was completed, and conclusions were drawn before it was revealed which group received spinal anesthesia and which received PNB.

## 3. Results

**Figure 2:**
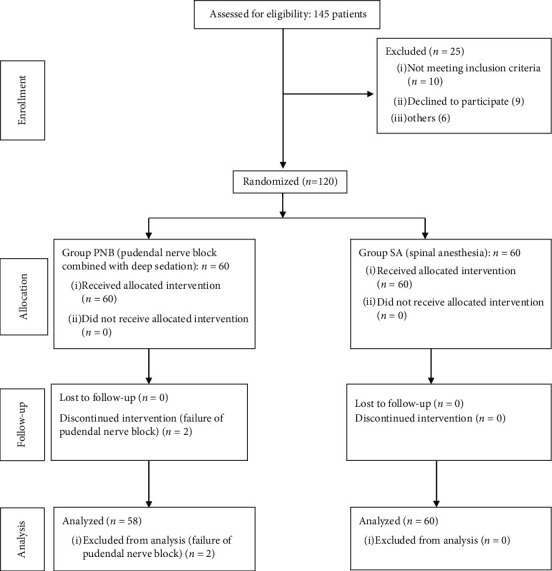
Consolidated standards of reporting trials flow diagram.

A total of 120 patients with hemorrhoids planned for elective Milligan–Morgan hemorrhoidectomy were enrolled in the study. The CONSORT diagram for patient recruitment is shown in Figure 2. Two patients were excluded after randomization because of the failure of PNB. There were no statistically significant differences in the patient demographics, the severity of the hemorrhoids, and the mean duration of surgery in both groups (Table 1).

Intraoperatively, five patients in Group SA and six patients in Group PNB developed hypotension and improved by a single intravenous injection of 6 mg ephedrine. The incidence of intraoperative hypotension had no difference between the two groups (8.3% vs 10.3%, *p*=0.707). Two patients had respiratory depression in Group PNB during operation and were alleviated by lowering the concentration of propofol. A laryngeal mask was unneeded to control ventilation.

Pain intensity was evaluated using VAS by two blinded hospital staff members. Patients in Group PNB had lower VAS scores (*p* < 0.001) at rest at 3, 6, 12, 24, 36, and 48 h and during mobilization at 12, 24, 36, and 48 h after surgery compared to the Group SA (Table 2).

Postoperative sufentanil consumption was another important indicator of postoperative pain intensity. The dosage of sufentanil for rescue analgesia was significantly lower in Group PNB compared to Group SA on day 1 (median, 12.0 vs 23, *z* = 7.209, *p* < 0.001) and day 2 after surgery (median, 6.0 vs 14.0, *z* = 7.760, *p* < 0.001) (Table 3). The time for the first rescue analgesia from the PCA device was significantly longer in Group PNB compared to Group SA (median, 14 vs 3.0 h, *z* = −9.27, *p* < 0.001) (Table 3).

The time to ambulation was significantly earlier in Group PNB compared to Group SA (median 2.0 vs 4.0 h, *z* = 7.973, *p* < 0.001) (Table 3). The first defecation occurred approximately 24 hours after surgery, and no statistical difference was found between the two groups (median, 22.0 (7) vs 22 (5) h, *z* = 0.339, *p*=0.735). However, the pain intensity at the first defecation was significantly increased in Group SA compared to Group PNB (median, 5.0 vs 4.0, *z* = 5.235, *p* < 0.001) (Table 3).

The anal relaxation played a vital role in the surgical conditions. No significant differences were observed in anal sphincter relaxation between the two groups (*p*=0.467) (Table 3). There was no difference in surgeon satisfaction with operative conditions between the two groups (*p*=0.148) (Table 3).

The patients in Group PNB had a lower incidence of postoperative urinary retention (6.9% vs 20.0%, *p*=0.034) and nausea and vomiting (5.2% vs 18.3%, *p*=0.023) compared to Group SA (Table 3). No significant differences were observed in the incidence of pruritus and respiratory depression after the operation in the two groups (Table 3). Patient satisfaction was significantly higher in Group PNB than that in Group SA (3.6 ± 1.0 vs 1.5 ± 0.5, *p* < 0.001).

## 4. Discussion

In this randomized controlled study, we demonstrated that ultrasound-guided bilateral PNB combined with deep sedation using propofol can successfully be applied to Milligan–Morgan hemorrhoidectomy. This anesthesia technique provided the same anal sphincter relaxation and surgical condition for surgeons as spinal anesthesia using 0.5% ropivacaine. Bilateral PNB was associated with better postoperative pain relief, reduced dosage of rescue analgesics, and lowered pain intensity at the first defecation. Moreover, PNB combined with propofol sedation could decrease the incidence of urinary retention, promoting early ambulation of patients compared to spinal anesthesia.

Spinal anesthesia was a conventional anesthesia method for hemorrhoidectomy, which could provide good muscle relaxation and inhibit visceral reflex. The patient tolerated surgery well under spinal anesthesia with mild sedation by dexmedetomidine. A loading dose before continuous infusion of dexmedetomidine is always recommended for sedation. However, five micrograms of sufentanil were intravenously injected for analgesia and sedation before spinal anesthesia in this study. Moreover, the length of the hemorrhoidectomy is about one hour in the authors' institution. Therefore, the patients were given the dexmedetomidine at the speed of 0.5 microg.kg^−1^.h^−1^ in Group SA, and the total dose of dexmedetomidine was about 0.5 microg.kg^−1^ in each patient. In addition, the patients in Group PNB also received PNB with 0.4% ropivacaine containing 0.5 microg.kg^−1^ dexmedetomidine. Therefore, maybe the patients were given the same dose of dexmedetomidine, which would affect the pain intensity after surgery.

Postoperative pain of hemorrhoidectomy mainly comes from the surgical incision and edema of perianal skin and mucosa [19]. Since perineum is an extremely sensitive region, the patients who underwent hemorrhoidectomy always experienced severe postoperative pain. Good pain relief can be achieved with caudal or spinal anesthesia, but the duration of analgesia is short lived and is frequently associated with some side effects especially urinary retention [9, 20]. Compared with conventional spinal anesthesia, saddle anesthesia has some advantages, such as less anesthetic dosage, lower block level, and faster recovery of motor function. However, saddle anesthesia still could not provide a long postoperative analgesia duration. Local infiltration could relieve postoperative pain in patients after hemorrhoids surgery, but the analgesia duration was just about 5–12 hours [4, 21, 22]. In this study, the results suggested that bilateral PNB could significantly decrease the pain intensity compared to spinal anesthesia. Moreover, the mean time for first rescue analgesia was 14 hours in the PNB group, and it was 4 hours in the SA group. These results were similar to previous studies reporting that PNB had a longer analgesic effect than local infiltration [5, 13]. In order to prolong the duration of PNB and provide better postoperative analgesia, dexmedetomidine was added as an adjuvant to the ropivacaine. The PNB may be an excellent method for pain relief after hemorrhoids surgery. However, PNB alone could not inhibit the vagal reflex during the operation, and deep sedation by target-controlled propofol was given in this study. Target-controlled infusion is the most commonly recommended method for intravenous anesthesia or sedation in China, especially when propofol is used. The LOC of propofol is an important parameter when TCI was used. The LOC in Chinese was about 2.2–2.3 microg.ml^−1^, and it should be maintained higher than that by about 20%–30% during the surgery according to the guideline of China. However, the LOC of propofol may be different in different races. We suggest that the LOC of every patient should be recorded when the target-controlled infusion is used.

Somatic nerve supply to the pelvic floor and external sphincters comes from sacral plexus (L4–L5 and S1–S4 segments). Pudendal nerve (S2–S4) is the main somatic nerve to provide the sensation of perineal skin and motor innervation to perineal muscles and external anal sphincter [23, 24]. Naja et al. [25] reported that patients could complete hemorrhoidectomy under nerve stimulation-guided PNB with either no sedation or mild sedation with only midazolam. Nevertheless, there were several differences between Naja et al.'s study and ours. Firstly, four points of injection and higher volume of anesthetic were given to the patients in their study. High volume anesthetics maybe not only block the pudendal nerve but also induce local infiltration anesthesia in perianal tissue. Secondly, Naja and his colleges used 2% lidocaine for PNB. A higher concentration of local anesthetic may provide better muscle relaxation, but this invisible puncture carried a high risk of injury to blood vessels, bowel, bladder, and other pelvic organs. In addition, the anorectal area was innervated by somatic and autonomic nerve together [26]. Deep sedation was adequate for mucosal dissection above the dentate line and inhibition of visceral reflex in Milligan–Morgan hemorrhoidectomy.

Urinary retention was another complication after hemorrhoidectomy. Previous studies have reported that the incidences of urinary retention after hemorrhoidectomy varied from 30.8% to 70% [3, 20]. There were many factors, including anesthesia, postoperative pain, volume of fluid administration, and surgery associated with urinary retention. In our study, the incidence of urinary retention in Group SA was 20.0%. On the other hand, the incidence of urinary retention in Group PNB was significantly reduced to 6.9%. Similar findings were recorded by a previous study; Anannamcharoen et al. found a higher incidence of urinary retention in the spinal anesthesia (30.3%) than those in the local anesthesia group (8.8%) [3]. Spinal anesthesia can interrupt the micturition reflex and causes vesical function disorders [27]. Vesical function disorders remain until the blockade is reduced to the third sacral segment [27]. Therefore, spinal anesthesia or epidural anesthesia plays a vital role in urinary retention. As PNB did not affect the micturition center, a previous study demonstrated that the addition of PNB to general anesthesia did not increase the incidence of urinary retention [12]. Postoperative pain is another vital factor in inducing urinary retention. It can stimulate the sympathetic nerve and reduce the tension of the detrusor muscle whose contract can promote urination [28]. Excellent postoperative analgesia may reduce postoperative urinary retention. Moreover, Tsai et al. [29] indicated that pudendal nerve blockade improved voiding function by decreasing external urethral sphincter hypertonicity in spinal cord injury patients. The inflammation of pelvic floor tissue caused by surgery and postoperative pain can also increase external urethral sphincter hypertonicity. Pudendal nerve blockade may promote urination by decreasing postoperative pain and external urethral sphincter hypertonicity.

Transperineal and transvaginal approach with or without nerve stimulator were two traditionally pudendal nerve blocked methods. However, these traditional approaches carried a high risk of injury to blood vessels, bowel, bladder, and other pelvic organs [30] Ultrasound-guided nerve block may allow direct visualization of the anatomical landmarks in close relationship with the pudendal nerve, such as the ischial spine, obturator muscles, and internal pudendal artery. Furthermore, the spread of the anesthetic solution can possibly be observed with real-time ultrasound. Therefore, ultrasonography may improve the precision and safety of the technique and minimize complications. The location for blocking the pudendal nerve was another issue deserving attention. Some studies block the pudendal nerve in the plane between sacrospinous and sacrotuberous ligaments at the ischial spine level by ultrasound- [31] and CT-scan-guided technique [32]. However, blockage of the pudendal nerve at the level of the ischial spine was associated with a risk of sacral plexus blockade [33]. In our early clinical practice, sciatic nerve blockade has also occurred when PNB was performed in this position. Moreover, a recent study showed that there was no significant difference in the accuracy of PNB between a blockade at the level of the ischial spine and the Alcock canal [34]. Therefore, in this study, we blocked the pudendal nerve at the entrance of the Alcock canal.

A previous study indicated that nerve trunks with a diameter less than 4 mm would not be detectable utilizing ultrasound with a 3.5 MHz curved-array probe at a depth of more than 5 cm [35]. The diameter of pudendal nerve ranged from 1.3 to 6.8 mm [35]. therefore, nearly one-half of the pudendal nerve could not be detected by ultrasound. Bellingham et al. showed that the pudendal nerve was sonographically visible only in 57% of the patients [33]. In our study, pudendal nerves were blocked by an injection of 0.4% ropivacaine around the internal pudendal artery under the guidance of ultrasound. We also utilized a nerve stimulator in PNB procedure. However, the stimulator was performed to avoid intraneural injection, not to guide nerve blockade, since the pudendal nerve has a poor response to the nerve stimulator possibly owing to the size of the nerve [31]. Moreover, injection of local anesthetics around the internal pudendal artery can successfully block the pudendal nerve [16].

In some medical institutions, hemorrhoidectomy is a daytime operation. General anesthesia is always performed combined with muscle relaxants which may increase the risk of postoperative respiratory depression. Moreover, general anesthesia requires higher medical costs to patients in some countries. Spinal anesthesia is another well used anesthesia technique for hemorrhoidectomy, but it increases the incidence of postoperative urinary retention. Bilateral PNB combined with deep sedation, which provides early ambulation, long postoperative pain relief, and low incidence urinary retention, may be a suitable anesthesia method for daytime operation with hemorrhoidectomy. However, further studies are needed to assess the feasibility and safety of this procedure for daytime operations.

There are a few limitations to this study. Firstly, complete blinding trials could not be performed because two different anesthesia techniques were utilized. However, the data collector for this study was blinded to the techniques used. Secondly, obese patients were excluded from the study because respiratory depression was more likely to occur when deep sedation was performed. Whether this anesthesia technique is suitable for these patients needs further study. Thirdly, the LOC of propofol of patients was not recorded in this study. In addition, the LOC may be different in different races, which is worthy of further study.

## 5. Conclusions

We conclude that pudendal nerve block using 0.4% ropivacaine combined with deep sedation using propofol can be successfully applied to Milligan–Morgan hemorrhoidectomy. In addition, a pudendal nerve block can provide better pain relief and a lower incidence of urinary retention after surgery compared to SA.

## Figures and Tables

**Table 1:** Demographic data and duration of surgery.

Patient characteristics	Group SA (*n* = 60)	Group PNB (*n* = 58)	*p*-value
Female/male	33/27	35/23	0.557
Age, *y* (SD)	41 (11)	40 (10)	0.742
Weight, kg (SD)	58.7 (8.7)	56.3 (7.8)	0.109
Height, cm (SD)	162 (6.5)	161 (6.8)	0.310
ASA status, I/II	35/25	30/28	0.472
Grades of hemorrhoids (III/IV), no. of patients	34/26	32/26	0.870
Operation time, minutes (SD)	43 (7)	44 (8)	0.306

Group SA: the patients received spinal anesthesia; Group PNB: the patients received bilateral pudendal nerve block combined with propofol sedation; ASA: American Society of Anesthesiologists; SD: standard deviation.

**Table 2:** Pain scores at 3, 6, 12, 24, 36, and 48 hours after surgery.

	Group SA (*n* = 60)	Group PNB (*n* = 58)	*p*-value
VAS scores at rest (0–10)			
3 h postoperatively	2.42 ± 1.22	1.03 ± 0.67	0.001
6 h	2.38 ± 1.12	1.08 ± 0.60	0.000
12 h	3.98 ± 1.19	2.09 ± 0.88	0.000
24 h	3.00 ± 1.04	2.03 ± 0.92	0.000
36 h	2.68 ± 0.93	1.88 ± 0.70	0.000
48 h	2.43 ± 0.85	1.50 ± 0.50	0.000
VAS scores on walking (0–10)			
12 h postoperatively	5.40 ± 1.33	3.38 ± 0.81	0.000
24 h	3.86 ± 1.26	2.62 ± 1.13	0.000
36 h	3.30 ± 1.01	2.17 ± 0.75	0.000
48 h	2.63 ± 0.90	1.79 ± 0.64	0.000

Values are shown as mean ± SD; Group SA: the patients received spinal anesthesia; Group PNB: the patients received bilateral pudendal nerve block combined with propofol sedation; VAS: visual analogue score.

**Table 3:** Secondary outcomes parameters.

	Group SA (*n* = 60)	Group PNB (*n* = 58)	*p*-value
Postoperative sufentanil consumption (*μ*g)			
Day 1, median (quartile range)	23 (12)	12.0 (6.5)	0.001
Day 2, median (quartile range)	14.0 (10.0)	6.0 (6.0)	0.001

The time for the first rescue analgesia, h, median (quartile range)	3.0 (2.0)	14 (7.3)	0.001
The time to ambulation, h, median (quartile range)	4.0 (2.0)	2.0 (1.0)	0.001
The pain intensity at the first defecation, median (quartile range)	5.0 (2.0)	4.0 (2.0)	0.001

Sphincter relaxation (*n*)			0.467
Full	45	40	
Incomplete	15	18	
No relaxation	0	0	

Surgeon satisfaction (*n*)			0.148
Terrible	0	0	
Satisfactory	5	10	
Excellent	55	48	

Urinary retention, *n* (%)	12 (20)	4 (6.9)	0.034
Nausea and vomiting, *n* (%)	11 (18.3)	3 (5.2)	0.023
Pruritus, *n* (%)	0 (0)	0 (0)	NS
Respiratory depression after surgery, *n* (%)	0 (0)	0 (0)	NS

*n*: number of patients. Group SA: the patients received spinal anesthesia; Group PNB: the patients received bilateral pudendal nerve block combined with propofol sedation; NS: not significant.

## Data Availability

The data used to support the findings of this study are included in the article.
